# A retrospective analysis of drugs associated with the development of cutaneous squamous cell carcinoma reported by patients on the FDA’s adverse events reporting system

**DOI:** 10.1007/s00403-024-03109-7

**Published:** 2024-05-25

**Authors:** Philippe Jean-Pierre, Keyvan Nouri

**Affiliations:** Phillip Frost Department of Dermatology and Cutaneous Surgery, University of Miami, 1150 NW 14th Street, Suite 500, Miami, FL 33136 USA

**Keywords:** General dermatology, Skin cancer, Squamous cell carcinoma, Adverse events, Side-effects, Medications, Drugs, Clinical research

## Abstract

Cutaneous squamous cell carcinoma (cSCC) is the second most common type of skin cancer arising from squamous cells of the epidermis. Most cases of cSCC have a good prognosis if detected and treated early; however, certain cases can be aggressive. The primary risk factor for cSCC is prolonged ultraviolet radiation from sun exposure, leading to DNA mutations. Other risk factors have also been observed, including adverse reactions to medications, particularly immunosuppressants. A query of the Food and Drug Administration Adverse Events Reporting System (FAERS) was done, and all reported events of cSCC as adverse events to medication were recorded along with demographic data of patients affected. A total of 4,792 cases of cSCC as an adverse event to medication were reported between 1997 and 2023. Lenalidomide, a chemotherapeutic drug, had the most cases of cSCC as an adverse event. Nine of the top 10 drugs associated with cSCC had immunosuppressive characteristics. While males had higher odds of cSCC associated with corticosteroids and calcineurin inhibitors, females had higher odds of cSCC related to monoclonal antibodies. Geriatric patients accounted for the majority of cSCC cases at 59.7%. Drawing on data from the FAERS database, there’s been a consistent increase in cSCC cases as a side-effect to certain medications, with most having immunosuppressive characteristics. Since there is a lack of up-to-date literature overviewing the most implicated medications for cSCC, we aimed to illustrate this better, as well as patient demographics, to better guide clinicians when prescribing these medications.

## Introduction

Cutaneous squamous cell carcinoma (cSCC) is a type of skin cancer that arises from epidermal squamous cells, typically due to DNA damage of cells leading to a malignant transformation [1]. cSCC is the second most common skin cancer, representing about 20 to 50% of all skin cancers in the United States [2]. While most cSCC are not life-threatening and can be treated with Mohs surgery or surgical excision if detected early, specific subsets of cSCC have more aggressive features associated with increased chances of metastasis and death [3].

Like other cutaneous malignancies, cumulative ultraviolet (UV) radiation from sun exposure is the most common risk factor for developing cSCC, hence why rates are highest in the elderly and fair-skinned individuals with minimal sun-protective melanin [4]. While cSCC can occur on any part of the skin, it most commonly presents on sun-exposed areas such as the head, neck, and arms. Although the most common cause of cSCC is cumulative UV exposure, other risk factors include exposure to arsenic and alkylating agents, oncogenic human papillomavirus, and immunosuppression [5–8]. Immunosuppressive agents increasing the risk of cSCC have been well established in the literature. Solid organ transplant recipients who require chronic immunosuppression have their risk of cSCC increased up to 65 to 250 times that of the general healthy population [9].

There needs to be more comprehensive literature assessing all the specific immunosuppressive agents that increase the risk of developing cSCC. Starting in 1993, the Food and Drug Administration (FDA) created a publicly accessible database, FAERS (Food and Drug Administration Adverse Event Reporting System that contains reports on adverse events suspected to be caused by medications. This database has improved the FDA’s ability to monitor approved drugs’ safety over extended periods. It aids clinicians in evaluating the association between medications and specific adverse reactions. In this report, we aim to explore medications and demographic information associated with the development of cSCC, as reported in the FAERS.

## Materials and methods

All adverse events labeled “Squamous Cell Carcinoma of Skin” were searched on the FAERS database. Reported adverse events linked to cSCC were collected, and a statistical analysis was performed. We tallied the aggregate number of adverse events associated with drugs most typically linked with cSCC to gauge its prevalence against other side effects. Cases reported that did not include the age and sex of patients were not included in our demographic analyses. Using patient sex as the primary variable, we used logistic regression to determine odds ratios (ORs). We generated ORs for corticosteroids, including prednisone, dexamethasone, and prednisolone, in the first analysis. We evaluated two monoclonal antibodies, adalimumab and infliximab, for the second analysis. Our third analysis evaluated two calcineurin inhibitors, tacrolimus and cyclosporine.

## Results

**Fig. 1:**
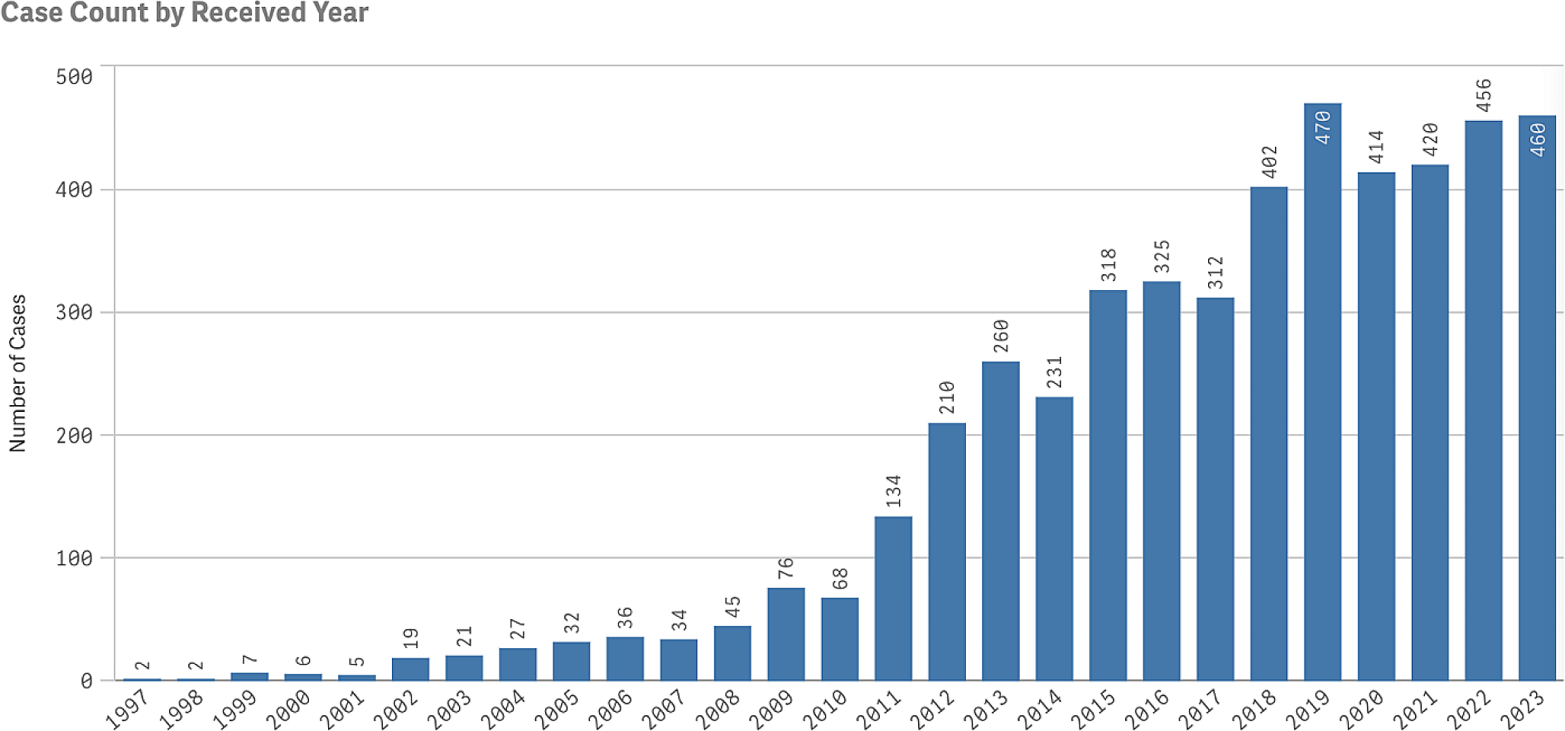
Reported cases of cutaneous squamous cell carcinoma as an adverse event

**Fig. 2:**
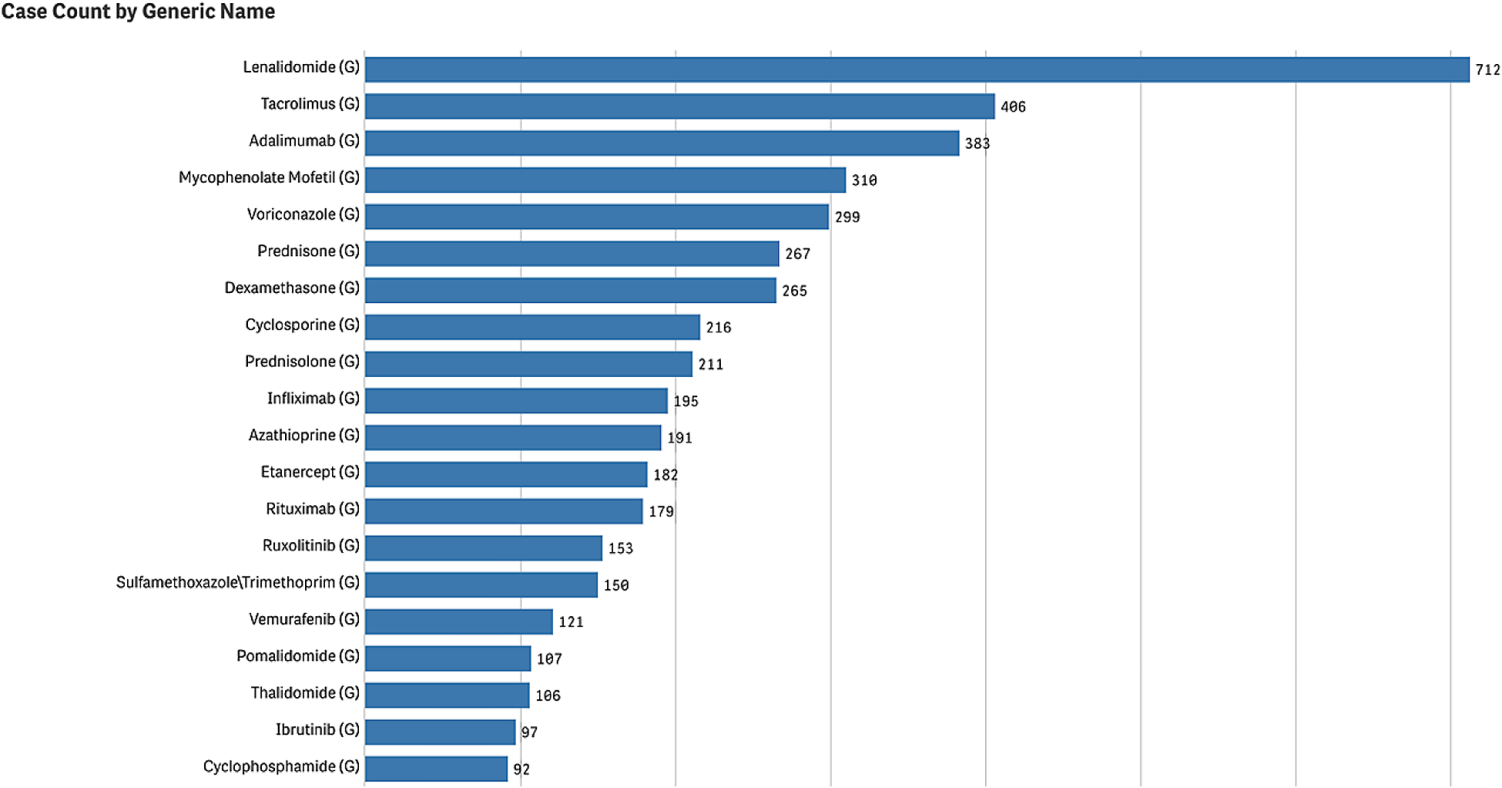
The top 20 medications associated with cutaneous squamous cell carcinoma as an adverse event

The FAERS database was queried on January 7th, 2024, and there were a total of 4,792 reported cases of cSCC as adverse events from medications. The distribution of adverse events of cSCC is displayed by year in Fig. 1, tracing the first report of cSCC back to 1997. Since then, there has been an apparent increase in reports, peaking at 470 cases in 2019. In Fig. 2, we highlight the twenty drugs most frequently implicated in reports of cSCC as an adverse event. Within the top ten drugs, lenalidomide is the most common at 712 (14.9%), followed by tacrolimus (8.5%), adalimumab (8.0%), mycophenolate mofetil (6.5%), voriconazole (6.2%), prednisone (5.6%), dexamethasone (5.5%), cyclosporine (4.5%), prednisolone (4.4%), and infliximab (4.1%). Nine of these ten medications had immunosuppressive components, and only voriconazole, an antifungal, did not.

Three of the drugs were categorized as corticosteroids, two were monoclonal antibodies, and two others were calcineurin inhibitors. Squamous cell carcinoma constituted 0.18% of all adverse events associated with prednisone, 0.27% for dexamethasone, and 0.27% for prednisolone. Our analysis found that males had higher odds of developing cSCC as a side effect from corticosteroids: prednisone (OR: 1.19, *P* < 0.05), prednisolone (OR: 1.54, *P* < 0.05), and dexamethasone (OR: 2.30, *P* < 0.05).

Among the top two monoclonal antibodies, cSCC represented 0.06% of all adverse events from adalimumab use and 0.10% for infliximab. Contrasting with corticosteroids, we found that cSCC as an adverse event from monoclonal antibodies were more likely to present in females: adalimumab (OR: 2.17, *P* < 0.05) and infliximab (OR: 1.55, *P* < 0.05). The top two calcineurin inhibitors, tacrolimus, and cyclosporine, had 0.39% and 0.26% cases of cSCC as a proportion of all adverse events, respectively. Like corticosteroids, males were more likely to experience cSCC as an adverse event: tacrolimus (OR: 1.85, *P* < 0.05) and cyclosporine (OR: 2.03, *P* < 0.05).

**Fig. 3:**
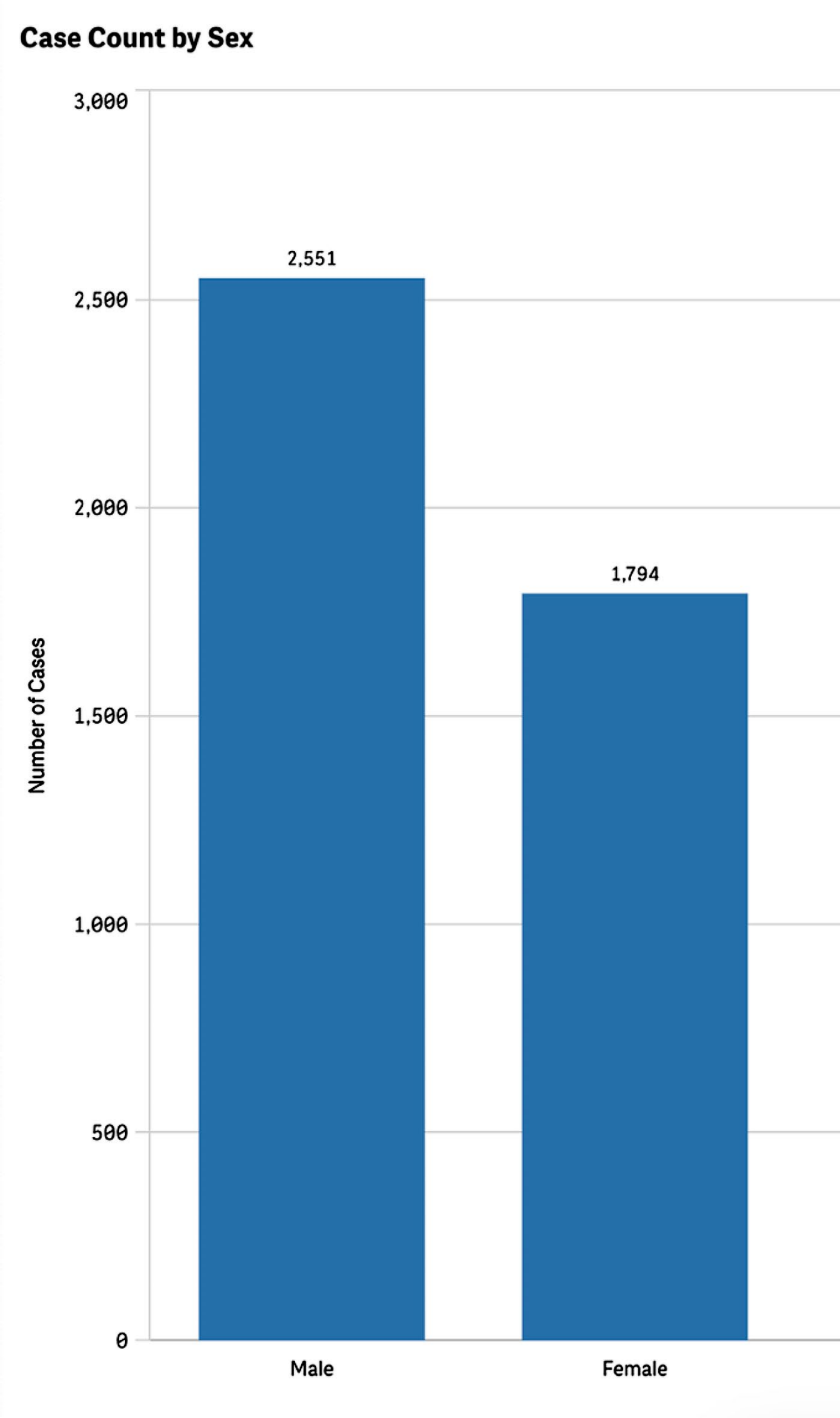
Reported cases of cutaneous squamous cell carcinoma by patient sex

**Fig. 4:**
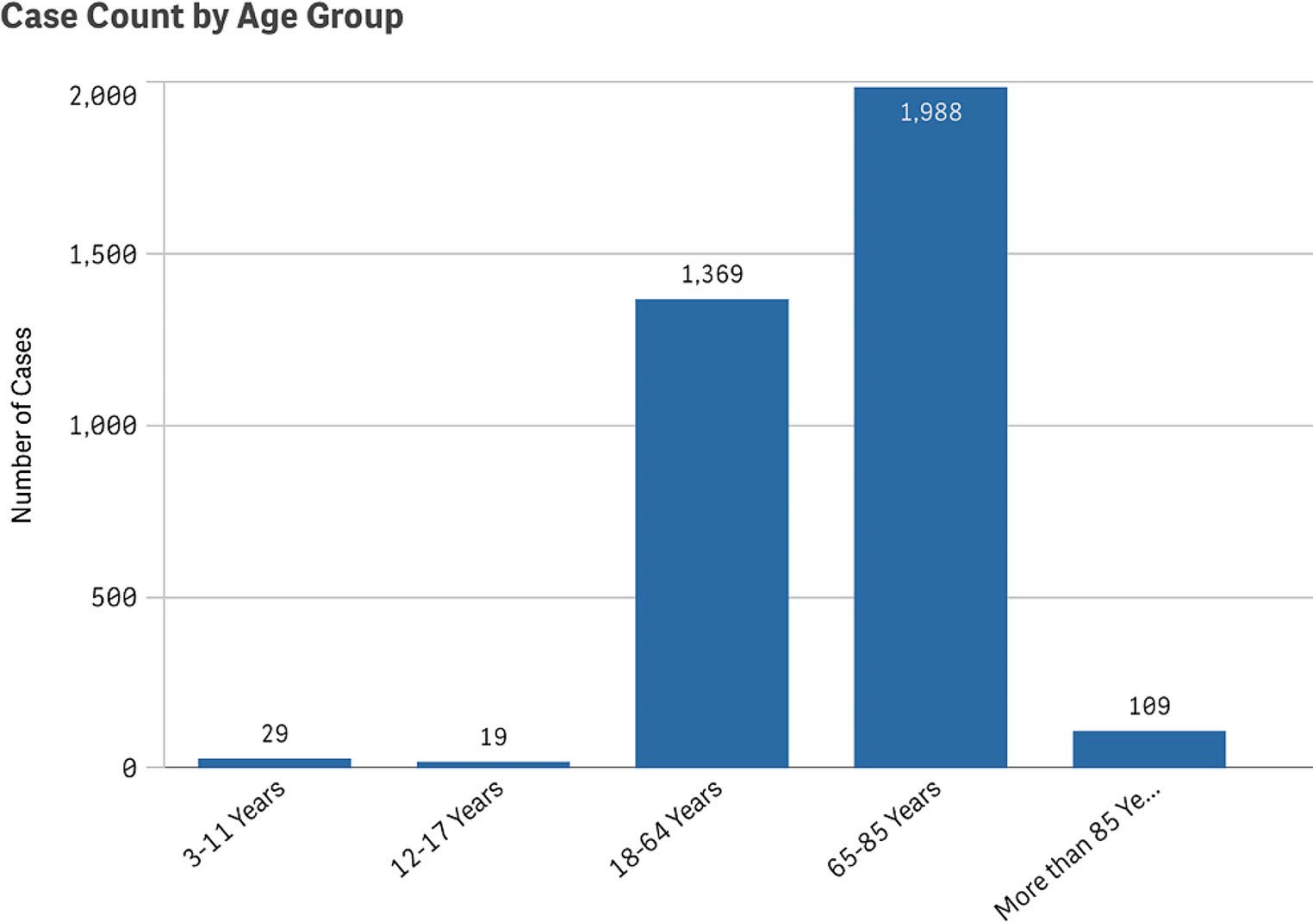
Reported cases of cutaneous squamous cell carcinoma by patient age group

Additionally, we analyzed patient demographics and found that most reported cSCC cases occurred in males at 58.7%, compared to 41.3% in females (Fig. 3). There were cases of cSCC reported as an adverse event in all age groups. However, the proportion of cases reported among pediatric patients was low at 1.37%, whereas elderly patients over the age of 65 represented 59.7% of all cases (Fig. 4).

## Discussion

This investigation provides an in-depth analysis of medications and patient demographics linked to the development of cSCC as an adverse reaction using the FAERS database. The FDA’s database has cataloged over 28 million adverse events over the years, with cases of cSCC only making up 0.017% of these, underscoring that it is relatively infrequent. Nonetheless, beginning in 2011, there has been a sharp increase in the number of cSCC reports to the FAERS database, which coincides with the rates of cSCC increasing in the general population. (10–11) As managing many debilitating diseases relies on immunosuppressive medications, clinicians and researchers must understand epidemiological trends and rates of adverse events associated with these drugs to assess their safety and better guide decisions when prescribing regimens.

The association of immunosuppression and increased risk of cSCC is well established in the literature. However, the precise mechanism by which this occurs is incompletely understood. Immunocompetence is vital in preventing cancer as the immune system can identify and eliminate cells that have undergone malignant transformation due to DNA damage [12, 13]. Thus, taking immunosuppressive drugs blunts this protection and permits cancer cells to proliferate more easily. This is consistent with our findings in the FAERS database analysis, as 17 of the top 20 medications associated with cSCC have immunosuppressive characteristics. Although these drugs have a relatively low rate of cSCC as an associated adverse event compared to other side effects, it is important to highlight these as patients who develop cSCC secondary to immunosuppressive regimens have been found to have more aggressive and rapidly progressing variants of cSCC. (14–15)

Two of the top 20 medications implicated in the development of cSCC did not have immunosuppressive properties: voriconazole and vemurafenib. The pathophysiology of voriconazole-associated cSCC is not well understood. Some suggested mechanisms include direct phototoxicity from voriconazole metabolites and the upregulation of COX-2, an essential enzyme in developing cSCC [16–18]. Vemurafenib is a BRAF inhibitor that is typically used to treat metastatic melanoma; however, this molecular mechanism can trigger RAS mutations due to a paradoxical activation of mitogen-activated protein kinase (MAPK), which can lead to the development of cSCC [19–21].

Cases of cSCC in the general population indicate that it is more prevalent in males than females [22]. However, this is likely because men spend more time exposed to the sun without adequate protection [23, 24]. Similarly, our investigation found that reports of cSCC as an adverse event to medications were overall most prevalent amongst males; however, in certain medication classes, such as monoclonal antibodies, cSCC was more prevalent in females. We highlight the need for further investigations into gender-based variations in medication adverse events, as this could help better elucidate which patients may be at higher risk of developing cSCC from certain drug classes.

There are a few limitations present in this study. Although the FDA regulates it, the FAERS database allows patients to self-report adverse events. Thus, we must consider the potential for erroneous reports, as the medical knowledge of the general public may vary significantly. Furthermore, some of the adverse events reported are cases in which the patients are taking multiple medications. Thus, there is the potential for these to serve as confounding factors. Lastly, some of the patients being treated with the reported drugs may have underlying medical conditions that may already predispose them to the development of cSCC.

## Data Availability

No datasets were generated or analysed during the current study.
